# Defining the hierarchical organisation of collagen VI microfibrils at nanometre to micrometre length scales^☆^

**DOI:** 10.1016/j.actbio.2016.12.023

**Published:** 2017-04-01

**Authors:** Alan R.F. Godwin, Tobias Starborg, Michael J. Sherratt, Alan M. Roseman, Clair Baldock

**Affiliations:** a Wellcome Centre for Cell-Matrix Research, Faculty of Biology, Medicine and Health, University of Manchester, M13 9PT, UK; b School of Biological Sciences, Faculty of Biology, Medicine and Health, University of Manchester, M13 9PT, UK

**Keywords:** Collagen VI, Articular cartilage, Pericellular matrix, SBF-SEM, Tomography

## Abstract

Extracellular matrix microfibrils are critical components of connective tissues with a wide range of mechanical and cellular signalling functions. Collagen VI is a heteromeric network-forming collagen which is expressed in tissues such as skin, lung, blood vessels and articular cartilage where it anchors cells into the matrix allowing for transduction of biochemical and mechanical signals. It is not understood how collagen VI is arranged into microfibrils or how these microfibrils are arranged into tissues. Therefore we have characterised the hierarchical organisation of collagen VI across multiple length scales. The frozen hydrated nanostructure of purified collagen VI microfibrils was reconstructed using cryo-TEM. The bead region has a compact hollow head and flexible tail regions linked by the collagenous interbead region. Serial block face SEM imaging coupled with electron tomography of the pericellular matrix (PCM) of murine articular cartilage revealed that the PCM has a meshwork-like organisation formed from globular densities ∼30 nm in diameter. These approaches can characterise structures spanning nanometer to millimeter length scales to define the nanostructure of individual collagen VI microfibrils and the micro-structural organisation of these fibrils within tissues to help in the future design of better mimetics for tissue engineering.

**Statement of Significance:**

Cartilage is a connective tissue rich in extracellular matrix molecules and is tough and compressive to cushion the bones of joints. However, in adults cartilage is poorly repaired after injury and so this is an important target for tissue engineering. Many connective tissues contain collagen VI, which forms microfibrils and networks but we understand very little about these assemblies or the tissue structures they form. Therefore, we have use complementary imaging techniques to image collagen VI microfibrils from the nano-scale to the micro-scale in order to understand the structure and the assemblies it forms. These findings will help to inform the future design of scaffolds to mimic connective tissues in regenerative medicine applications.

## Graphical abstract

**Figure:**
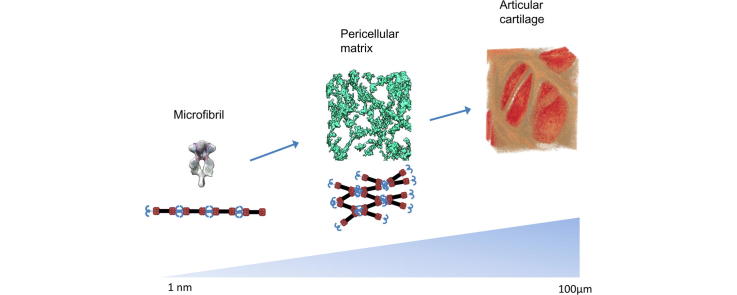

## 1 Introduction

Articular cartilage protects the articulating joints during movement; cartilage is hypocellular and is mainly composed of extracellular matrix [1]. In adults, cartilage is poor at repairing itself after trauma which can lead to degenerative cartilage diseases such as Osteoarthritis (OA). One method of repairing this damage would be to use tissue engineering to regenerate damaged cartilage by differentiating stem cells, such as adipose-derived adult stem cells or mesenchymal stem cells, into chondrocytes using scaffolds which mimic the biomechanical properties of cartilage [2], [3]. This goal however, has proven difficult to achieve, with scaffolds not supporting chondrocyte maintenance often forming fibrous or fibrocartilaginous tissue instead of hyaline cartilage [4]. It is therefore important to understand the structural and mechanical properties of the matrix surrounding chondrocytes to create better biomaterials to stimulate cartilage regeneration. A key component of the PCM surrounding chondrocytes in articular cartilage is collagen VI which is important for maintaining the biochemical and mechanical properties of cartilage [5], [6], and has been shown to enhance cartilage tissue regeneration [7].

Collagen VI is a unique collagen that forms beaded microfibrils that are structurally distinct to fibrillar collagens and the substructure within them also termed “microfibrils”. Unlike the fibrillar collagens, collagen VI forms heteromeric microfibrils with a beads-on-a-string appearance [8]. Collagen VI is found in most connective tissues including articular cartilage, kidney, tendon, cornea and skin, where unlike the other microfibrillar assembly (fibrillin) it is resistant to *in vivo* photoageing and the action of UV radiation *in vitro* [9]. However, mutations in collagen VI mainly affect the muscular skeletal system leading to diseases such as Bethlem myopathy, Ullrich’s congenital muscular dystrophy and OA [10], [11]. Collagen VI acts as an anchor between cell surface receptors, such as integrins [12], [13] and the matrix. Collagen VI has been shown to interact with a large number of matrix components such as; decorin, collagen II, collagen IV, aggrecan and fibronectin [14], [15], [16]. This link between the cell surface and the matrix has been shown to be cytoprotective as disruption of collagen VI causes an increase in apoptosis through perturbation of autophagy [17], [18]. Collagen VI is also a key component of the stem cell niche and through regulating matrix stiffness is involved in maintaining the ability of adult muscle stem cells to self-renew [19].

**Fig. 1:**
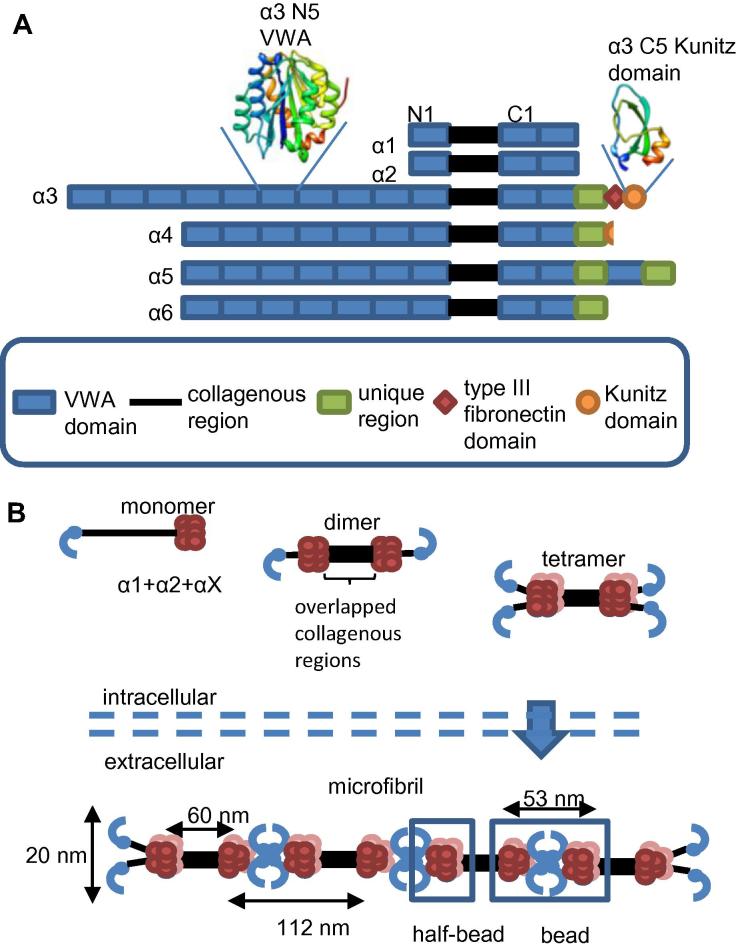
Domain organisation of collagen VI α-chains and microfibril formation. A) A cartoon illustrating the domain organisation of the collagen VI α chains. The VWA domains are numbered from N1 and C1 from the closest domains to the collagenous region. Also shown are cartoon representations of the structures of the α3 chain N5 VWA domain [53] and the α3 chain Kunitz domain [69]. The domain cartoons are rainbow coloured from blue at the N-terminus to red at the C-terminus. B) A cartoon representation of collagen VI microfibril assembly. Collagen VI heteromeric monomers form from an α1, α2 and αX chain where X can be α-chains 3–6. Triple-helical monomers then form disulphide linked dimers and then tetramers before being secreted into the extracellular space where microfibrils are formed. C-terminal globular regions are shown in red, N-terminal regions are shown in blue. The bead and half-bead regions of the microfibril are highlighted. The bead region contains the same number of VWA domains as a tetramer with the half-bead being equivalent to a dimer. The mature microfibril contains 10 C-terminal VWA domains in each half-bead [34]. (For interpretation of the references to colour in this figure legend, the reader is referred to the web version of this article.)

There are six collagen VI chains α1-6 [20], [21], [22], [23] with short collagenous regions flanked by globular N and C-termini, which are mainly composed of domains homologous to the A-domains of Von Willibrand factor (VWA) [20], [24] (Fig. 1). Chains α1 and α2 are similar in size and domain structure whereas the α3 chain is much larger [25]. The α4, α5 and α6 chains have similarities to the α3 chain, each of these chains contain 7 N-terminal VWA domains [21]. In humans and chimpanzees the α4 chain is not expressed but has been implicated in increased susceptibility to OA [26].

Three collagen VI α-chains associate to form heterotrimeric monomers [8], [27] which are stabilised by the formation of inter-chain disulphide bonds [27], [28] (Fig. 1). Collagen VI monomers form disulphide bonded homodimers then tetramers which are secreted into the extracellular space. The end-to-end assembly of tetramers [27] forms beaded microfibrils which have globular regions separated by triple-helical collagenous regions [8]. Collagen VI microfibrils do not form in the absence of the α1 chain [29] so it is likely that monomers are formed from one α1 chain, one α2 chain and a long chain (α3, α4, α5 or α6 chain) which can be interchangeable based on their similarity to each other [30]. The C-terminal domains of each chain are thought to be involved in chain association and selection [31] and at least 5 N-terminal VWA domains are required for microfibril assembly [32]. Collagen VI dimers and tetramers are homotypic containing only one type of long chain, however heteromeric microfibrils can form from different long chains [33].

A 3D model of the collagen VI bead region generated using negative stain TEM [34] shows three distinct regions in each “half-bead” which have been termed the head, intermediate, and tail regions. The head region has a compact structure and is likely formed from the C-terminal VWA domains with the tail regions formed from the N-terminal VWA domains. The tail regions show a higher degree of heterogeneity than the homogenous head region suggesting a degree of flexibility. EM and SAXS studies of recombinant α3 N9-N1 region and α4, α5, α6 N1-N7 regions showed that the VWA domains form a very similar compact C-shape [33], [34]. The collagenous region in the microfibril forms a segmented twisted supercoil, predicted due to the imperfections in the repeating Gly-X-Y motif [35]. The collagenous regions of the *anti*-parallel dimer can be seen twisting round each other in TEM images, and bifurcation of the strands can be seen at the bead regions [36]. However, many aspects of the molecular organisation are still undefined for example the conformation of the tail regions and whether they are involved in microfibril assembly, or interactions with other matrix proteins.

Collagen VI microfibrils form larger networks in a tissue specific manner. In skin, an irregular web-like network of collagen VI microfibrils associates with collagen II and III fibrils. Collagen VI fibres can be seen running parallel and in-between banded collagen fibrils [37]. In tissue culture, collagen VI can form bundles of aligned filaments which have ∼100 nm periodic banding [38]. These large banded structures can also be seen in diseased tissue [39], [40], [41]. *In vitro* assays have demonstrated that collagen VI tetramers can assemble to form large hexagonal networks when incubated with biglycan [42]. Hexagonal arrangements of collagen VI networks can also be observed in tissue culture [8] suggesting this could be a physiologically occurring structure but these have yet to be imaged in tissues. Collagen VI is highly expressed and widely distributed in the PCM surrounding chondrocytes in articular cartilage [6] where it plays a key role in the PCM structure. Correct organisation of the PCM is essential for maintaining mechanical properties of cartilage and for transducing biomechanical signals from the surrounding matrix to chondrocytes [5]. Immuno-fluorescence [43], and helium ion microscopy [44] show that the PCM is formed from a basket-like meshwork of matrix surrounding the chondrocyte but the connections made between microfibrils within this meshwork are still unresolved.

To determine the nanostructure of collagen VI and the organisation of the globular regions, we imaged isolated collagen VI microfibrils using cryo-TEM and created 3D reconstructions using single particle averaging techniques. Furthermore, to investigate the 3D micro-structure of collagen VI *in situ* in the chondrocyte PCM, murine articular cartilage was imaged using electron tomography and serial block face (SBF)-SEM imaging.

## 2 Material and methods

### 2.1 Tissue sources

Murine articular cartilage was extracted from 6 month old C57BL/6 db/- (Jackson Labs). Mice were sacrificed by asphyxiation using CO_2_ gas following home office guidance. Adult bovine eyes were obtained from a local abattoir.

### 2.2 Collagen VI microfibril extraction

Collagen VI microfibrils were extracted from bovine cornea using collagenase as described previously [34]. Approximately 0.2 g (wet weight) of bovine cornea was diced before being suspended in 2 ml of digestion buffer (400 mM NaCl, 20 mM Tris-HCl (pH 7.4)) which contained 0.1 mg/ml of chromatographically purified bacterial collagenase type VII (Sigma-Aldrich), and protease inhibitors (3 mM NEM, 5 mM PMSF). Digestions were incubated overnight at 4 °C whilst undergoing gentle stirring. Digested tissue was centrifuged for 3 min at 800*g* and the supernatant size fractionated on a Sepharose CL-2B column (GE Healthcare) equilibrated in 150 mM NaCl, 20 mM Tris-HCl, 2.5 mM CaCl_2_ (pH 7.4).

### 2.3 Sodium dodecyl sulphate polyacrylamide gel electrophoresis and western blotting

Collagen VI samples were subjected to SDS-PAGE under reducing conditions with 5% (v/v) β-Mercaptoethanol using 4–20% Mini-PROTEAN TGX precast gels (Biorad) and bands were visualised using Instant Blue (Expedion). Western blotting used polyclonal rabbit anti-collagen VI antibody (NB120-6588 (Novus Biologicals) 1:1000) and goat anti-rabbit antibody conjugated with horse radish peroxidase (Dako) (1:3000). Bands were visualised using enhanced chemiluminescence (Interchim) and imaged using the ChemiDoc imaging system (Biorad).

### 2.4 Cryo-transmission electron microscopy

Collagen VI samples were adsorbed onto glow discharged 0.2 μm holey carbon Quantifoil 2/2 grids, which had been coated with a thin layer (∼2 nm) of carbon. Samples were adsorbed for 1 min before washing with water. Grids were blotted and plunge frozen in liquid ethane using a FEI Vitrobot plunge freezer. Blot times ranged between 2.5 and 5 s and the Vitrobot was maintained at 4 °C and at 95% humidity. Samples were imaged at −170 °C under low dose (∼20 e^−^/Å^2^) conditions on a FEI Tecnai G2 Polara TEM operating at an accelerating voltage of 200 kV at a magnification of 39000× which resulted in a sampling of 3 Å/pixel. Images were collected using a Gatan Ultrascan 4000 CCD camera with a defocus range of −2 to −5 μm.

### 2.5 3D reconstruction of collagen VI microfibrils

Collagen VI bead regions (particles) were manually boxed using a 256 × 256 pixel box in the EMAN-2 software suite [45] resulting in a dataset of 1060 particles. Particles were corrected for the contrast transfer function (CTF) by phase-flipping before being edge-mean normalised and low pass filtered to 20 Å using a top-hat filter using the program SPIDER [46]. Particle stacks were iteratively rotationally and translationally aligned using the local projection matching program FindEM [47], [48] to a template reference image. This was iterated over 26 rounds, with the template updated to be the new average of aligned particles after each round. To avoid reference bias the initial template was the average image of the unaligned stack of particles. A binary mask which covered the collagen VI bead region was drawn using the program WEB [46]. A half-bead particle stack was generated from the aligned particles as described previously [34]. An initial 3D model was then constructed by creating a cylindrical model from the sum-average of the particle set using SPIDER. The sum-average was back projected using a simultaneous iterative reconstruction technique (SIRT) based method and C-100 symmetry imposed. The initial model was then iteratively refined using a projection matching procedure implemented using FindEM and SPIDER. The initial model was projected at an angular increment of 5° around the fibre axis to give an angular sampling sufficient for 20 Å resolution for an object of this size. The model projections were used as references for multi-reference based rotational and translational alignment using FindEM. After the orientation of particles was assigned based on cross correlation to the model projections, particles with the same orientation were summed into class averages and back projected using SIRT in SPIDER to construct a new model. This procedure was repeated until a stable model was generated. During the iterative refinement 2-fold symmetry was imposed along the fibre axis of the model as previously described [34].

### 2.6 Atomic force microscopy (AFM)

Collagen VI microfibrils (25 μl) were adsorbed onto ethanol washed 1.5 mm glass coverslips for 1 min before washing with ddH_2_O. Coverslips were imaged using a Multimode 8 AFM, with scan assist tip, whilst operating in ScanAssyst air mode (Brucker). Images were processed using Nanoscope v8.15 (Brucker). Individual beads were selected with a 78 nm × 78 nm box and were analysed using the ImageJ measure tool. The median intensity value for the whole image was subtracted from the volume measured for each bead to give a final, background-subtracted, bead volume [9].

### 2.7 Sample preparation for electron tomography and serial block face scanning electron microscopy (SBF-SEM)

Murine articular cartilage samples were prepared for electron tomography and SBF-SEM as previously described [49]. Briefly, cartilage samples were fixed in 2.5% (v/v) glutaraldehyde and 4% paraformaldehyde (w/v) in 0.1 M cacodylate buffer for 24 h. Samples were then decalcified, to prevent damage to the sectioning knife, by incubation in 14% EDTA at 4 °C for 7 days with the EDTA solution changed daily. Samples were further fixed and stained for 1 h in 1% osmium tetroxide and 1.5% Potassium ferrocyanide (w/v) in 0.1 M cacodylate buffer. Samples were then treated with 1% (w/v) tannic acid in 0.1 M cacodylate buffer for 1 h, after which samples were washed in distilled water before further staining in 1% (w/v) osmium tetroxide. After osmium staining, samples were washed before incubation in 1% (w/v) uranyl acetate for 1 h. Samples were then dehydrated in a series of alcohol dilutions (50–100%) and in acetone before embedding in TAAB 100 hard resin (Agar Scientific).

### 2.8 Electron tomography

Thick sections (∼250 nm) were cut from samples embedded in resin using a Diatome diamond knife and Leica ultramicrotome. Sample sections were mounted on formvar carbon coated copper slot grids (Agar Scientific) and 10 nm colloidal gold solution was applied to both sides. Single axis tilt series were taken from −65° to +65° in 1° steps using a FEI Tecnai G2 Polara TEM operating at an accelerating voltage of 300 kV, at a magnification of 23000X. Images were collected using a Gatan Ultrascan 4000 CCD camera using the software SerialEM [50]. The tilt series of images were aligned and tomograms were generated by back projection in IMOD using the Etomo workflow [51].

### 2.9 Serial block face scanning electron microscopy

Murine articular cartilage samples were imaged at a magnification which resulted in a sampling of 10 nm/pixel using the Gatan 3view system (Quanta FEG 250 (FEI) equipped with a Gatan3View ultramicrotome) using a 3.8 kV accelerating voltage and 0.4 Torr chamber pressure. Primary electron back-scatter was used to image the block face after each section of 100 nm was removed from the surface. A data set of 314 images was collected.

## 3 Results

### 3.1 Collagen VI microfibril single particle averaging and 3D model reconstruction

**Fig. 2:**
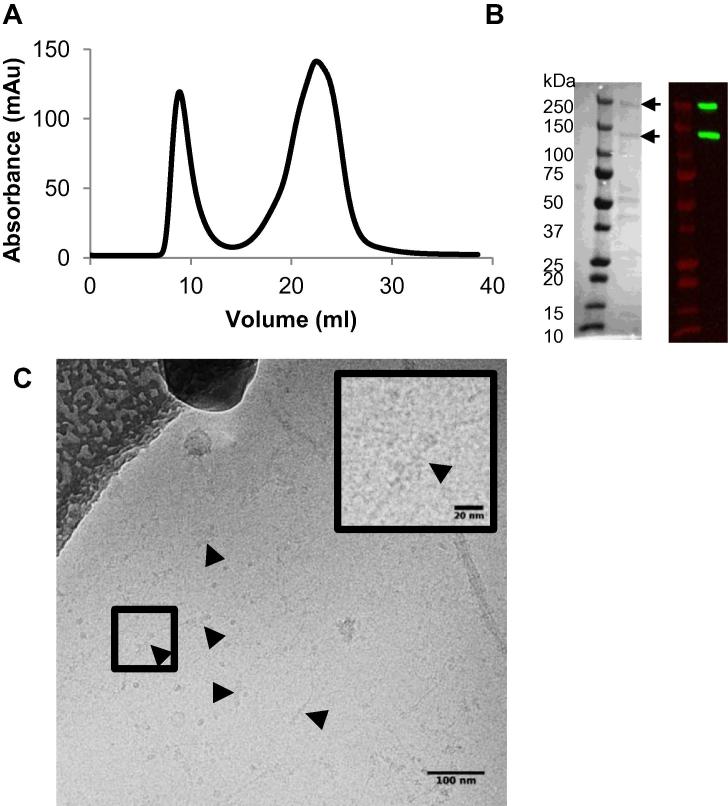
Purification and imaging of collagen VI microfibrils from bovine cornea. (A) Size exclusion chromatography of collagenase extracted bovine corneal tissue using a Sepharose Cl-2B column. The absorbance (mAu) at 280 nm is plotted against the elution volume (ml). The first peak represents the void volume of the column where microfibrils elute. (B) Reducing SDS-PAGE (left hand panel) and western blot (right hand panel) of the central fraction of the void peak. Collagen VI chains were detected using a polyclonal rabbit anti collagen VI antibody. Arrows highlight bands at approximately 250 kDa, which corresponds to the α3 chain, and at 120 kDa which corresponds to α1 and α2 chains. C) Bovine collagen VI was imaged under cryo conditions using a FEI Tecnai G2 Polara TEM operating at an accelerating voltage of 200KV. Black arrows highlight the globular bead regions. A magnified image of a bead region is shown in the top right of the figure.

Previous 3D reconstruction of collagen VI using negative stain-TEM revealed the organisation of C- and N-terminal VWA domains in the bead region [34], but the N-terminal regions were not resolved in this structure, potentially due to flexibility or to staining/dehydration issues. Therefore to eliminate the latter sources of potential artefacts, tissue extracted microfibrils were purified for cryo-TEM and frozen in a hydrated state. Collagen VI microfibrils were purified from bovine cornea using enzyme digestion and size exclusion chromatography (Fig.2A). This tissue is readily available in sufficient quantities for microfibril purification and as there is high sequence conservation between human, bovine (86% identical to human) and mouse (81% identical to human) collagen VI, it represents a useful model source [34]. SDS-PAGE and western blotting of purified fractions showed two bands with sizes of ∼250 kDa and ∼130 kDa (Fig.2B). Previous mass spectrometry studies of extracted corneal collagen VI had identified the ∼250 kDa band as collagen VI α3 chain and the ∼130 kDa band as collagen VI α1 and α2 chains [34]. Purified corneal microfibrils were imaged using cryo-TEM; a representative micrograph is shown in Fig.2C. Microfibrils had the characteristic globular bead structures separated by the collagen helical region as has been seen previously using negative stain TEM [34] and rotary shadowing [52].

**Fig. 3:**
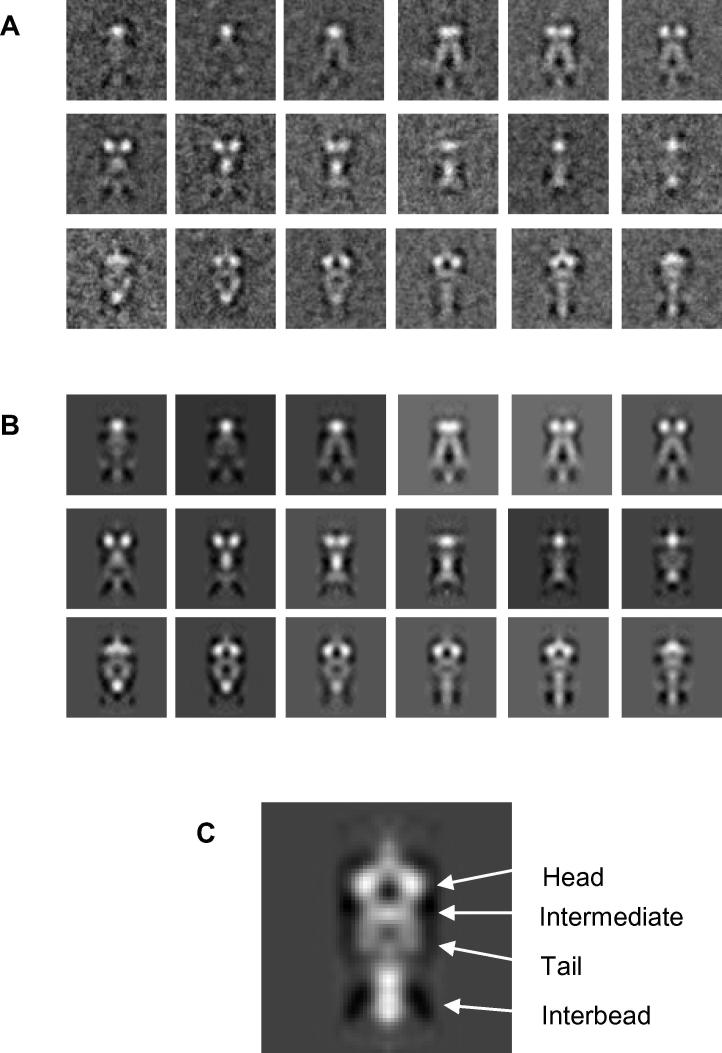
3D reconstruction of the collagen VI microfibril bead region. Individual images of collagen VI bead regions were cropped from cryo-TEM images using EMAN2 [45] and aligned using FindEM [48]. Half-bead particles were extracted from aligned stacks of beads before being reconstructed into a 3D model using single particle reconstruction methods using FindEM and SPIDER [46]. (A) Class-sum images of aligned particles. Particles were classified by similarity to model projections using cross-correlation. (B) Reprojections of the final half-bead model. Class-sum images and model reprojections represent 10° rotations around the collagen VI fibre axis. (C) The central slice from a radial average of the collagen VI half-bead model. The box size is 77 × 77 nm for all panels.

Micrographs were analysed using the program EMAN2 [45] and 1060 bead regions from the collagen VI microfibrils were selected with a 256 × 256 pixel box size. Bead region images were corrected (normalised and CTF corrected) before being iteratively rotationally and translationally aligned to an average image of the unaligned data set to avoid reference bias using the software FindEM [48]. Due to flexibility within the bead region, “half-beads” were selected from the aligned collagen VI bead regions which are the equivalent to a dimer of the triple-helical collagen VI (Fig.1B). These half-beads were classified into classes containing similar orientations to generate classum images (Fig.3A). A model of the collagen VI half-bead was reconstructed using iterative model based refinement and back projected at 10° rotation intervals around the fibre axis (Fig.3B). Classum images show a well-defined head and intermediate regions, but have less well defined tail regions (Fig.3C). This is potentially due to heterogeneity caused by flexibility in the tail region. Classum images and the reprojections are highly similar showing the model is consistent with the aligned images. The radial average of the half-bead model shown in Fig.3C shows four distinct layers. The head region has dimensions of 6.6 nm × 13.2 nm; the intermediate region has dimensions of 4.1 nm × 12 nm and the tail regions 9 × 6 nm which are similar to the dimensions of previously published negative stain TEM reconstructions [34].

**Fig. 4:**
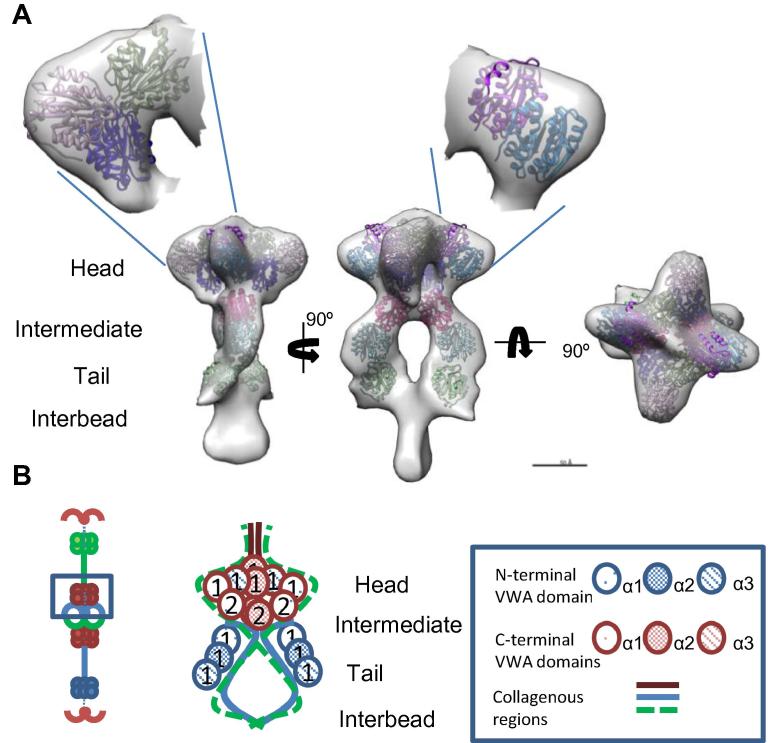
3D structure of collagen VI microfibril. (A) Cryo-TEM structure of collagen VI visualised using UCSF Chimera [54]. VWA domains were placed in the electron density map by hand before their fit was optimised using the UCSF Chimera fit in map tool [54]. Ten C-terminal VWA domains were fitted into the head region and 3 VWA domains were fit in the intermediate and tail regions. (B) Schematic model of the organisation of VWA domains in the half-bead structure. Three VWA domains were fitted in each of the larger lobe-like structures, potentially corresponding to the VWA C1 and C2 from either α1 or α2 chain and C1 from the α3 chain, and each of the smaller lobes could accommodate the remaining two VWA domains from either the α1 or the α2 chain. The intermediate and tail region could accommodate three VWA domains which could correspond to the N1 VWA domains from the α1, 2 and 3 chains.

The head region is composed of four lobe-like structures which connects to the intermediate region, and two C-shaped tail regions joined by the collagenous interbead region (Fig. 4). The resolution of the collagen VI half-bead reconstruction was estimated by calculating the Fourier Shell Correlation (FSC). The FSC is calculated by comparing the 3D Fourier transforms of two models calculated from two halves of the data set. The model had an estimated resolution of 48 Å at the 0.5 FSC threshold (Supplementary Fig. 1). The α3 chain N5 VWA domain [53] was used to model the VWA domains in the half-bead (Fig. 4). The domains were placed into the half-bead model by hand before the fit was iteratively optimised using the UCSF Chimera fit in map tool [54] using the same method as previous described [34]. Ten C-terminal VWA domains could be fitted into the head region, this included three VWA domains in each of the two larger lobe-like structures, and each of the smaller lobes could accommodate two VWA domains. As a half-bead has an equivalent number of VWA domains as a dimer this is consistent with the C1 and C2 VWA domains from each of the α1 and α2 chains and one C1 VWA domain from each α3 chain (Figs. 1B and 4B), which has been shown previously [34]. The two larger lobe-like structures could contain the C1 and C2 domains from either α1 or α2 chain and the C1 from the α3 chain (it has previously been shown that C2-C5 domains from the α3 chain are cleaved off [34]). Each of the two smaller lobes could accommodate the remaining two VWA domains from either the α1 or the α2 chain, although in this model it is not possible to specify which α chain is in each lobe. A model for the organisation of the VWA domains is shown in Fig.4B. The tail region is likely formed from the N1 domains from α1, 2 and 3 chains and these regions could accommodate six VWA domains consistent with a collagen VI triple-helical dimer in the half-bead structure. This new model is largely in agreement with the previous negative stain derived model [34]. A distinct difference though is the four lobed head structure, which was not seen previously. The frozen-hydrated preparation method used in this study is a much more reliable preservation method of molecular structures, and details of the four lobed feature may previously have been lost due to dehydration and distortion (flattening) caused by the negative staining preparation technique.

### 3.2 Atomic force microscopy

In the collagen VI half-bead reconstruction the N-terminal tail regions seem to be less well defined than the head region. This region was also shown to be poorly defined in the negative stain TEM model of the half-bead of collagen VI [34]. To determine if the heterogeneity observed in the N-terminal tail regions is due to flexibility in the α3 chain N-terminal VWA domains or potentially due to different compositions of α-chains, the volume of collagen VI beads was measured using AFM. Collagen VI microfibrils are formed from heterotrimeric monomers of α1 + α2 and αX (where x can be any one of the long α-chains 3, 4, 5, or 6), the α3 chain can also undergo alternative splicing where N-terminal VWA domains are spliced out [55].

**Fig. 5:**
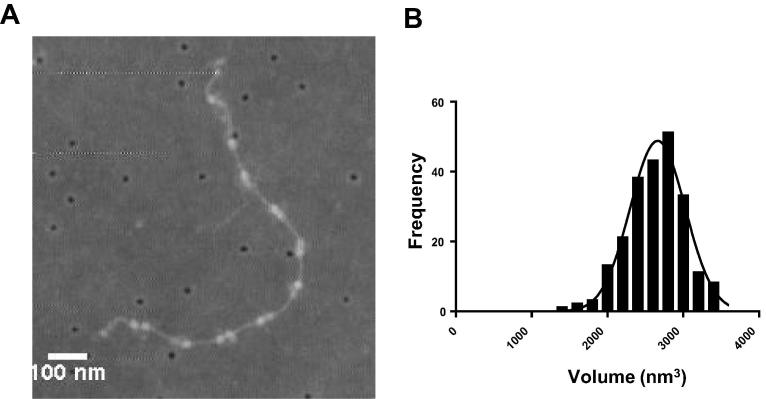
AFM analysis of collagen VI microfibrils. (A) An AFM image of isolated collagen VI microfibrils adsorbed onto a glass cover slip. (B) A histogram of collagen VI bead region volumes. A Gaussian curve was fitted to the data using non-linear regression in GraphPad Prism version 6.04. The data fit with an R square of 0.972 and had a calculated mean value of 2662 nm^3^. A total of 225 bead regions were measured.

Collagen VI microfibrils were adsorbed onto glass coverslips and imaged using AFM (Fig.5A). The volume of the bead region (n = 255) was measured using ImageJ and the background of the image was then subtracted. The maximum bead height was 4.64 nm and the mean microfibril bead volume was 2662 nm^3^ (Fig.5B). The data fits a single Gaussian distribution suggesting that a single α-chain variant is present.

### 3.3 3D reconstructions of murine chondrocyte pericellular matrix

Collagen VI is found in high concentrations in the PCM surrounding chondrocytes in articular cartilage, where it plays a key role in the PCM structure. The correct composition of the PCM is essential for maintaining the mechanical properties of cartilage and for transducing biomechanical signals from the surrounding matrix to chondrocytes [5]. Although collagen VI has an important role it is still not fully understood how microfibrils are organised in the PCM surrounding chondrocytes. To address this, electron tomography and SBF-SEM has been used to create 3D models of the chondrocyte PCM in murine articular cartilage.

### 3.4 Imaging the nanoscale structure of articular chondrocyte pericellular matrix using electron tomography

**Fig. 6:**
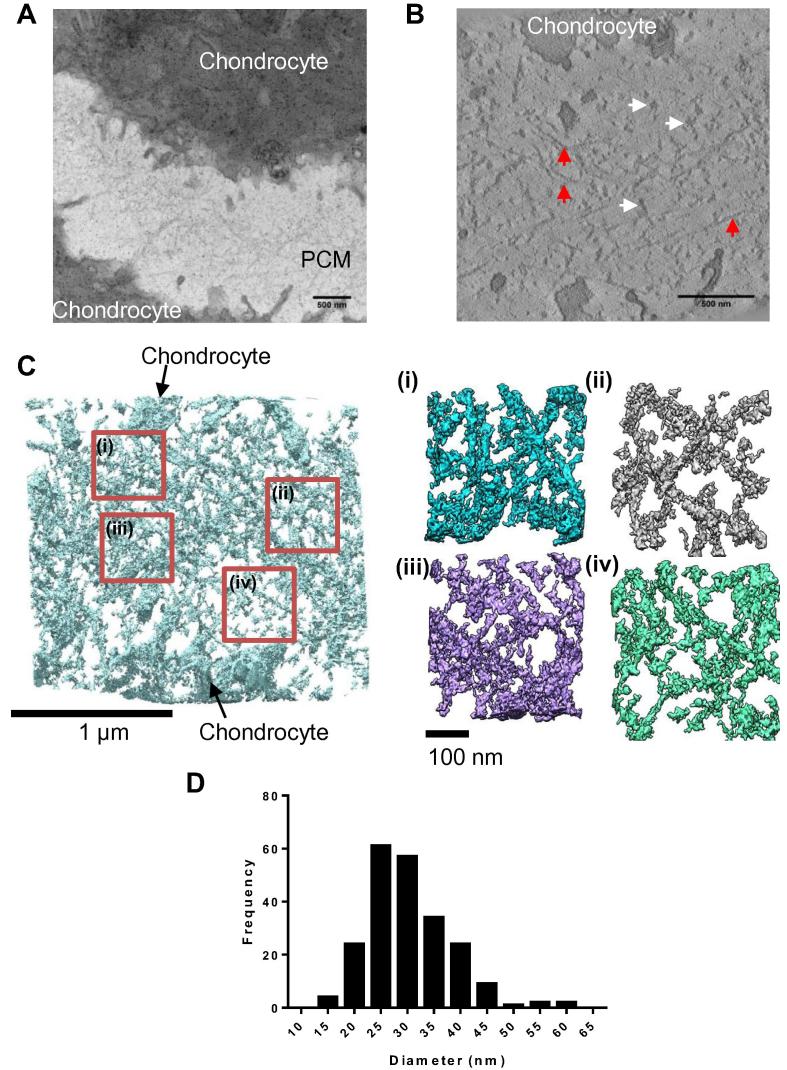
Murine articular cartilage electron tomography. Articular cartilage was imaged using TEM and a tilt series collected. (A) Representative image of the PCM between two chondrocytes. (B) A virtual z-slice from a tomogram of the chondrocyte PCM. Highlighted with white arrows are globular densities which are potentially bundles of collagen VI and red arrows indicate straighter fibrillar structures potentially collagen II fibrils (C) A tomogram rendered using UCSF Chimera. Red boxes define regions of interest magnified in panels i–iv. (D) Diameters of PCM globular densities were measured using ImageJ and plotted as a histogram of their diameters. The mean diameter was 30.4 nm ± 0.5 nm (SEM). A total of 218 particles were measured from one tomogram. (For interpretation of the references to colour in this figure legend, the reader is referred to the web version of this article.)

To determine how collagen VI is organised in the PCM, thick sections from murine articular cartilage were imaged using a FEI Tecnai G2 Polara operating at an accelerating voltage of 300 kV. From a low magnification image (Fig.6A), areas of interest were selected and electron tomography tilt series were collected from +65° to −65° at a magnification of 23000×. Tilt series were aligned and tomograms were reconstructed using back projection using the IMOD software suite [51]. A representative z-slice from a tomogram of the murine PCM is shown in Fig.6B. A network of globular structures can be seen spanning the gap between the two chondrocyte cell membranes (Fig.6B). The meshwork can also be seen to contact fibres, running parallel to the chondrocyte cell surface, which are likely collagen II due to their straight fibrillar appearance and relatively thin diameter (Fig.6B). The tomogram was rendered in 3D using UCSF Chimera (Fig.6C). Magnified volumes (Fig.6C(i–iv)) show a more detailed view of the meshwork showing the globular densities forming a network connected by thin fibrils. The program ImageJ was used to analyse the diameters of the globular densities; as tomograms had a low contrast and were relatively noisy, automated particle analysis could not be used so particles were hand segmented. Particles (n = 218) were measured from virtual z-slices from the tomogram; a histogram of the particle diameters is shown in (Fig.6D). Globular densities had a mean diameter of 30.4 nm ± 0.5 nm (SEM).

### 3.5 Microscale imaging of murine articular cartilage by serial block face scanning electron microscopy

**Fig. 7:**
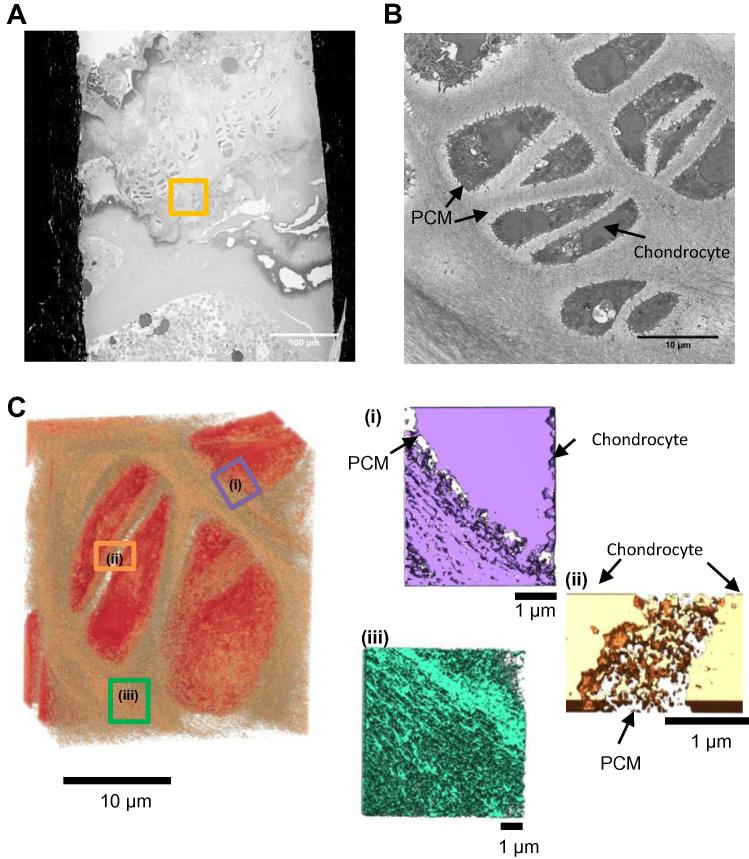
Murine articular cartilage imaged using SBF-SEM. Murine articular cartilage tissue was imaged using SBF-SEM. (A) An image of the sample block face is shown, highlighted is the region where the SBF-SEM data set was collected. (B) A representative image from the SBF-SEM data set where the PCM and chondrocyte are labelled. (C) 3D reconstruction of a sub-volume of the SBF-SEM data-set (dimensions of 27 × 23 × 16 μm), the right panel shows 3D reconstructions of the PCM surrounding chondrocytes (i and ii) and territorial matrix (iii).

The same sample block imaged by electron tomography was also imaged using SBF-SEM, allowing correlation of tissue structures from tens of nanometers to hundreds of microns. The microscope images the surface of a sample using back scattered electrons before a section from the sample is removed using an in-built microtome before imaging the surface again. Fig.7A shows an image of the sample block face; the region where the SBF-SEM data set was collected is highlighted. A data set of 313 images was collected with a sampling of 10 nm/pixel with sections of ∼100 nm thickness removed after each scan to image a ∼31 μm tall block of tissue. A representative image from the data set is shown in Fig.7B. The PCM can be seen as a light halo of less densely packed matrix surrounding the chondrocyte. Further from the chondrocyte the territorial matrix can be distinguished from the PCM as a more densely stained fibrillar matrix. The SBF-SEM data set was rendered in UCSF Chimera and several sub-regions of the volume are shown in Fig.7C. The PCM surrounding chondrocytes is shown in Fig.7C (i and ii). The PCM can be seen as a less dense region of matrix between the chondrocyte and the adjacent territorial matrix. The PCM between the two chondrocytes can be seen to be organised into a mesh like network which is very similar to the structure determined using electron tomography. Fig.7C(iii) shows a volume of the territorial matrix which in contrast to the PCM is formed from densely packed fibrils.

## 4 Discussion

Here we present the first 3D reconstruction of collagen VI microfibrils using cryo-TEM. The model has a compact hollow head region composed of four lobe-like structures which likely contain the ten C-terminal VWA domains from the three α-chains. The intermediate region connects the head region to the two tail regions which have a compact C-shape which could accommodate the N1 VWA domains from the three α-chains. The additional N-terminal domains from the long α3 chain are absent from the structure but present in the microfibrils as shown by SDS-PAGE. Therefore the loss of density is likely due to heterogeneity in this region caused by flexibility of these domains. Indeed, SAXS studies on recombinant N-terminal VWA domain arrays have shown them to be flexible [33]. The observed heterogeneity is less likely to be caused by different splice variants of the α3 chain as SDS-PAGE and AFM volume analysis of the bead region suggested that it was made up of a single species. Previous studies of bovine corneal collagen identified that collagen VI microfibrils were composed of VWA C1-N6+N8 from the α3 chain [34] and mass spectroscopy analysis did not identify α4, 5 or 6 chains. Therefore due to flexibility in this region it is still not clear how the N-terminal VWA domains are arranged in the bead region of the microfibrils. Reconstructions presented here could resolve part of the collagenous region which connects the two half-beads and which was poorly defined in previous negative stain studies [34]. It is likely that the collagenous regions go around the outside of the hollow head region but increasing the resolution of this structure, for example by utilising the more sensitive direct detection devices [56], may in future allow resolution of these regions.

**Fig. 8:**
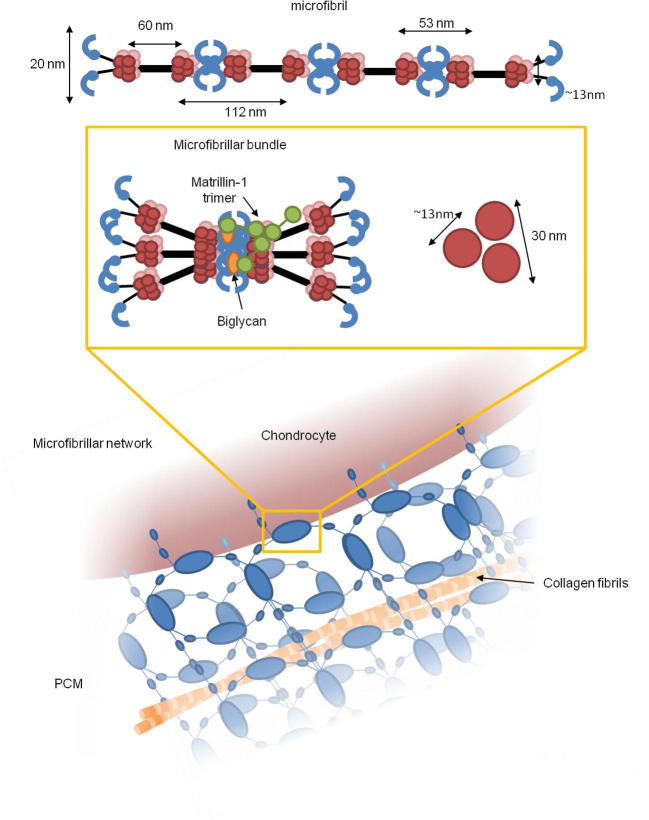
Model of collagen VI hierarchical organisation A collagen VI microfibril forms bundles potentially through interaction with adaptor complexes such as biglycan via the collagen VI N-terminal regions [58]. Shown in the right hand panel is a schematic diagram of a cross-section of a bundle of three microfibrils which are ∼13 nm in diameter, forming a complex ∼30 nm in diameter. Microfibrillar bundles can then form larger hexagonal networks in the PCM. The microfibrillar bundles become nodes which are connected by individual microfibrils.

The PCM between two chondrocytes in murine articular cartilage was imaged using SBF-SEM and electron tomography which allows for the 3D reconstruction of the tissue. The PCM was formed from a dense meshwork of globular densities which due to its distribution most likely consists of collagen VI microfibrils. The mesh-like network had a similar appearance to the meshwork observed using helium ion microscopy [44] and similar diameter to the hexagonal arrangements which form *in vitro* when purified collagen VI are incubated in the presence of biglycan [42]. The globular densities had a larger diameter than a single collagen VI microfibril and are likely to be multiple microfibrils complexed with other PCM molecules and adaptor proteins (Fig. 8). Collagen VI microfibrils may form microfibril bundles through interaction with their N-terminal tails with the small leucine-rich proteoglycans biglycan or decorin [42], [57]. These microfibril bundles can then from larger networks further facilitated by binding to other matrix proteins, such as collagen II in complex with the adaptor protein matrillin-1 [58], [59] and perlecan. Perlecan has been shown to colocalise with collagen VI [60] and mapping of the PCM mechanical properties show that perlecan and collagen VI colocalise in areas where the matrix is less stiff next to the chondrocyte [61]. So it is likely that perlecan also forms part of these assemblies in the PCM. Cell surface receptors such as integrins may also facilitate the organisation of collagen VI microfibrils into these larger networks. Collagen VI binds to α1β1, α2β1 and α10β1 integrins through direct interaction with its collagenous region [12], [13], [62], [63], [64], [65] this binding is not dependent on bridging molecules such as fibronectin [66] Studies using different chondrocyte cell lines have suggested that the primary integrin involved in collagen VI binding is α1β1 [64].

Collagen VI is a major component and plays a key role in defining the mechanical properties of the PCM surrounding chondrocytes. This has been demonstrated by force mapping which correlates the presence of collagen VI with a lower elastic modulus [67]. The mechanical properties of the PCM are therefore likely to be highly dependent on the structure of the collagen VI network. Imaging the structure of the collagen VI network will also be key in understanding how mechanics of the PCM change with disease [68]. The main form of collagen VI expressed in the PCM is thought to contain the α3 chain. The α6 chain is also expressed in articular cartilage but has a different expression pattern as it is expressed further into the territorial matrix and less in the PCM [21]. This raises the possibility that the α-chain composition of the collagen VI microfibril could be what defines the diameter of the PCM meshwork structure. The α6 chain like the α3 chain is widely expressed and is also found in tissues such as skin, lung and blood vessels, so it would be interesting to see how the overlapping expression of multiple α-chain variants affects collagen VI microfibril hierarchical structure in tissues.

## 5 Conclusion

In this study, we have imaged collagen VI at different levels of hierarchy and at different length scales from the 3D nanoscale structure of purified microfibrils to the microscale networks of collagen VI *in situ* in the PCM of articular cartilage. Understanding the tissue structure of collagen VI will give greater insights into the role of collagen VI in health and diseases such as OA as well as providing insights into the role of collagen VI in organising PCM structure. A greater understanding of these structures will also be useful in engineering better replacements for regenerative medicine applications.
