# Supplementary material for: Defining the hierarchical organisation of collagen VI microfibrils at nanometre to micrometre length scales
Source: Acta Biomater. 2017 Apr 1;52:21–32. doi: 10.1016/j.actbio.2016.12.023 (PMC5402720; doi:10.1016/j.actbio.2016.12.023)
Supplement: Supplementary data [file mmc1.pdf]

Supplementary figure 1

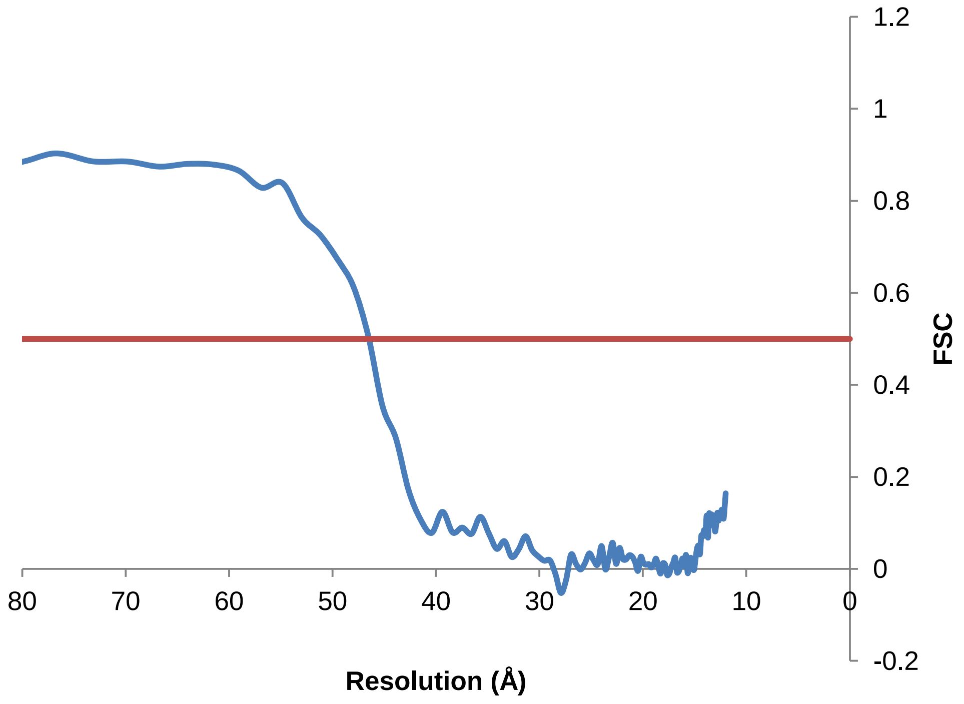

Supplementary Figure 1: Collagen VI half-bead resolution estimate
The resolution of the final model was calculated using FSC of two models reconstructed from two halves of the data set using SPIDER [46]. The FSC is plotted against spatial resolution. The resolution of the model was estimated, at the 0.5 FSC threshold shown as a red line, as 48 Å.
